# Anterior leaflet replacement and reconstruction with Admedus Cardiocel™ decellularized pericardial patch in tricuspid valve endocarditis

**DOI:** 10.1093/jscr/rjab106

**Published:** 2021-03-29

**Authors:** Saifullah Mohamed, Akshay J Patel, Khurum Mazhar, Ravish Jeeji, Paul D Ridley, Lognathen Balacumaraswami

**Affiliations:** 1 Royal Stoke University Hospital, Stoke On Trent, UK; 2 Institute of Immunology and Immunotherapy, University of Birmingham, Birmingham, UK

## Abstract

We present the case of a 28 year-old lady with a history of intravenous drug use who presented to our institution with symptomatic right heart failure secondary to tricuspid valve regurgitation. She presented with infective endocarditis leading to dyspnoea and peripheral oedema secondary to torrential tricuspid regurgitation. Transthoracic echocardiography confirmed right ventricular dysfunction and congestive hepatomegaly. Intra-operatively findings an infected and destroyed anterior leaflet of the tricuspid valve with posterior leaflet prolapse was found to cause severe tricuspid regurgitation. She had complex tricuspid valve reconstruction using anterior leaflet reconstruction using Admedus Cardiocel™ patch, posterior leaflet prolapse correction and commissural reduction with a McGoon imbrication and annuloplasty ring to stabilize the repair. This case demonstrates the importance of reconstructive tricuspid valve surgery in the setting of infective endocarditis. Furthermore, this case demonstrates the possibility of anterior leaflet excision and reconstruction with an excellent durable functional result.

## INTRODUCTION

De Vega [1] described tricuspid valve repair as a surgical procedure for tricuspid regurgitation in the 1970s. Newer techniques were described later and these include complex annular and leaflet reconstructive procedures [2]. A myriad of techniques such as tricuspid valvectomy, repair and replacement have been advocated depending on the aetiology and status of the patient at the time of the operative procedure. A recent meta-analysis and systematic review comparing tricuspid valvectomy with replacement in patients with intravenous drug use demonstrated it to be acceptable initial procedure with a staged replacement procedure following valvectomy in self-selected survivors [3]. Tricuspid valve reconstructive techniques have been described for congenital anomalies with absent leaflets [4], benign neoplasms affecting a single leaflet [5, 6], and in the setting of endocarditis [7–10] and the European Society of Cardiology and the American Heart Association [11, 12] have supported these. Furthermore, when considering aetiology limited to infective endocarditis different surgical approaches and techniques have been described. These include valvectomy [3], leaflet and annular reconstruction [4–8], use of mitral homografts partial/complete [9], and chordal repairs [10]. We describe an interesting case of a young woman with a history of intravenous drug use and tricuspid valve endocarditis who was referred for surgery.

## CASE PRESENTATION

We present the case of a 28 year-old lady with a history of intravenous drug use who presented to our institution with symptomatic right heart failure secondary to tricuspid valve regurgitation. She presented with infective endocarditis leading to dyspnoea, peripheral oedema and congestive hepatomegaly secondary to torrential tricuspid regurgitation. She abstained from intravenous drugs and had been maintained on methadone therapy prior to her surgery. Transthoracic echocardiography confirmed tricuspid valve endocarditis and severe tricuspid regurgitation with evidence of right ventricular dysfunction and congestive hepatomegaly. Based on positive serial blood cultures (*Staphylococcus aureus)* and echocardiographic findings, antibiotics were commenced (IV Flucloxacillin and IV Vancomycin) as soon as the diagnosis was made. Owing to the compromised clinical picture of the patient (haemodynamic instability and potential for life-threatening sepsis), urgent surgery took place after 2 days of full antibiotic therapy. Coronary angiography was normal. Despite the clinical and echocardiographic features of right heart failure, the patient remained in sinus rhythm.

**Figure 1:**
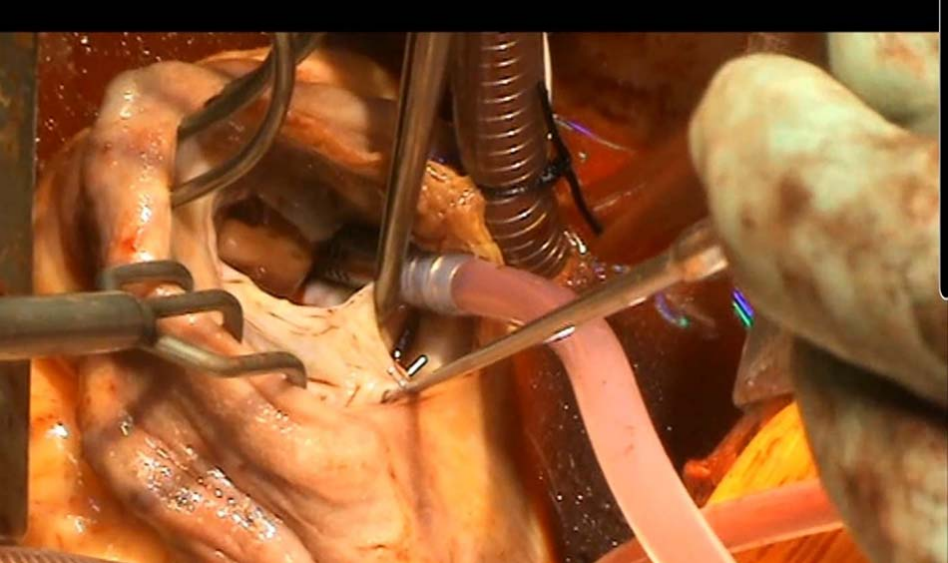
Anterior leaflet destroyed by vegetation and posterior leaflet prolapse.

Intra-operatively there was an infected and destroyed anterior leaflet of the tricuspid valve with posterior leaflet prolapse causing severe tricuspid regurgitation (Fig. 1). There was evidence of raised pulmonary artery pressures and right ventricular dysfunction. A detailed assessment of the remnants of the native tricuspid valve was performed on the operating table to devise a complex reconstructive strategy.

**Figure 2:**
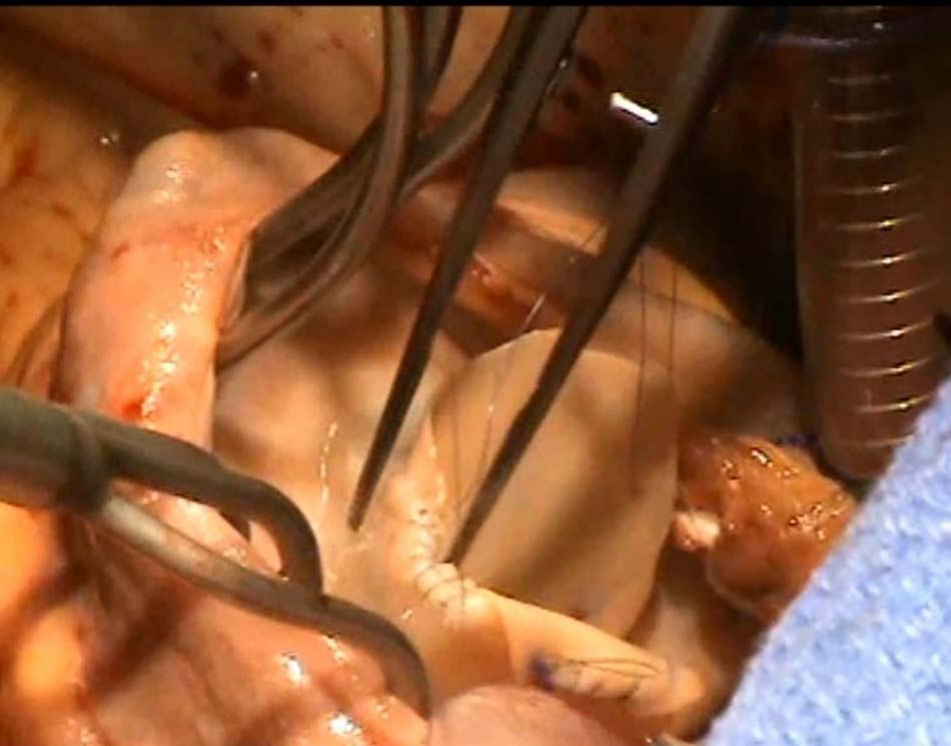
Reconstruction of the anterior leaflet with the Admedus decellularized bovine pericardial patch.

**Figure 3:**
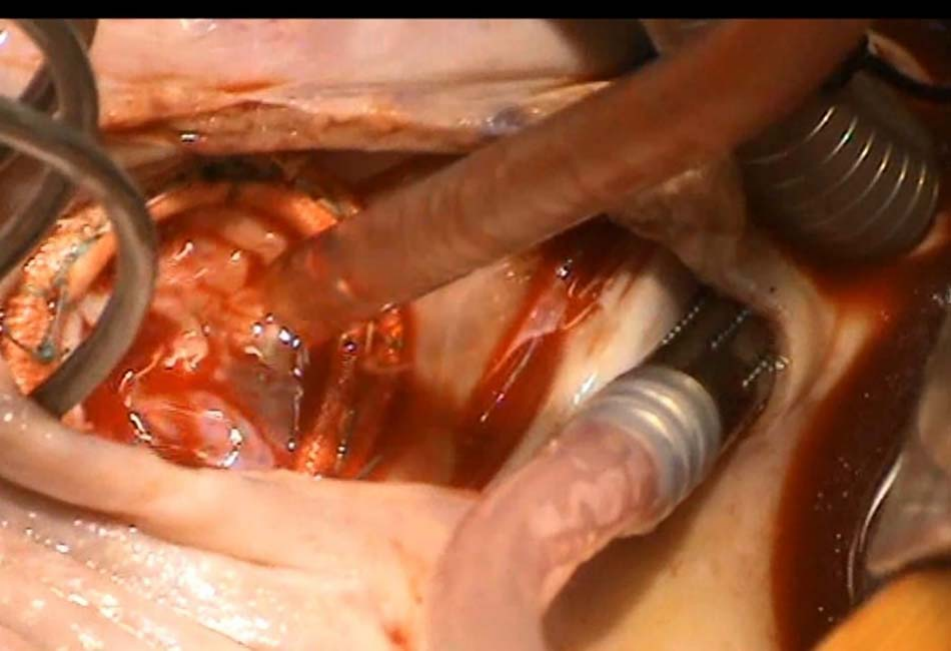
Demonstration of a competent tricuspid valve following complex tricuspid valve reconstruction

The anterior leaflet was almost totally destroyed by infection with only a rim of annular tissue. The posterior leaflet was prolapsing with a significantly dilated corresponding posterior annulus and the septal leaflet was tethered with corresponding annular dilatation. The anterior leaflet was reconstructed using Admedus Cardiocel™ decellularized bovine pericardium (Fig. 2). The template for the anterior leaflet was derived based on the space required with an additional 25% facilitating anchoring of the chordae to the neo leaflet. A 30-mm Medtronic Tri-Ad Adams Tricuspid® annuloplasty ring sizer provided an adequate template. Using 6′0″ polypropylene sutures in a semi-continuous fashion, the patch was carefully sewn ensuring adequate provision for redundant tissue along the annular margin to prevent tension at the sutured margin. Three distinct areas were marked for anchoring of free margin 5′0″ Goretex neochordal sutures and proximally to the corresponding papillary muscle bases along the anterior and posterior leaflets and thirdly onto the *trabeculated* free right ventricular wall between the two papillary muscle bases. The neochords were placed with figure of eight stitches on the papillary muscle and free wall and passed through the free margin of the neo anterior leaflet. These were left loose to be subsequently adjusted after annuloplasty ring placement. The septal leaflet was sized along its base and a downsizing annuloplasty using a 26-mm Medtronic Tri-Ad Adams Tricuspid® annuloplasty ring was chosen ensuring that the sutures in the septal area were into the base of the leaflet to avoid complete heart block. The annuloplasty ring was implanted using interrupted 3′0″ Cardioflon sutures. Subsequently, the neo anterior leaflet neochords were adjusted to the height at which the free margin would be level with the base of the septal leaflet to ensure correct coaptation. Static testing was performed and two aspects required further correction. The posterior leaflet medial edge was prolapsing and this required correction by approximation to the lateral margin of the neo anterior leaflet, which achieved an optimal height of the posterior leaflet to facilitate coaptation without any leaflet restriction or prolapse (Fig. 3). There was obvious prolapse of the medial aspects of both septal leaflet and the medial remnant of the anterior leaflet which were brought to the correct coaptation height using multiple Cardionyl sutures and a McGoon imbrication stitch.

Post-bypass transoesophageal echocardiography demonstrated a stable repair with no valvular regurgitation. Following discharge, the patient progressed well to having a durable repair at 2 years following surgery with improvement in symptoms and complete resolution of the hepatic congestion and heart failure and she remained in sinus rhythm.

## DISCUSSION

Tricuspid valve reconstructive surgery (TVRS) for infective endocarditis remains a contentious topic of debate amongst the cardiac surgical community. TVRS in contrast to mitral valve repair is complicated by the impact of the dynamics of right ventricular contraction and response of the subvalvular apparatus to the variable loading conditions of the right heart. Hence, TVRS has been performed infrequently and limited to the anterior tricuspid valve leaflet [7]. We describe a standardized technique of complex tricuspid valve reconstruction that allows the surgeon to devise an appropriate zone of coaptation and adjust the height of the free leaflet margin. This case demonstrates the feasibility of anterior leaflet excision and reconstruction using the Admedus™ decellularized bovine pericardial patch to produce an excellent and durable functional result. This strategy avoids mechanical tricuspid valve replacement, which necessitates anticoagulation and is associated with a very high incidence of artificial prosthesis-related complications.

## CONFLICT OF INTEREST STATEMENT

None declared.

## FUNDING

None.
